# The coronavirus disease 2019 (COVID-19) pandemic in nursing homes – the experience of care workers in Poland

**DOI:** 10.12688/f1000research.124984.1

**Published:** 2022-09-07

**Authors:** Ryszard Necel

**Affiliations:** 1 Department of Sociology, Adam Mickiewicz University, Poznan, 60-568, Poland

**Keywords:** nursing homes, social care, care workers, culture of disaster, mental health

## Abstract

**Background: **Nursing homes in Poland are the most common formal care institutions for dependent people. During the coronavirus disease 2019 (COVID-19) pandemic, nursing homes had particularly high infection rates. In this context, it is important to ask about the experiences of the care workers working in these institutions.

**Methods: **This research was conducted using the computer-assisted web interviewing (CAWI) technique in five provinces in Poland. The field research was carried out in April 2021. The research sample included, among others, nurses, care workers, therapists, social workers and the management staff of institutions whose representatives worked during the COVID-19 pandemic. Respondents were asked to assess the care provided to residents.

**Results: **It turned out that the vast majority of respondents positively assessed the fulfillment of the basic living needs of residents and the availability of care. The assessment of the organization of residents’ leisure time, the fulfillment of their religious and cultural needs, and the issue of maintaining contacts with the social environment was less satisfactory. The article also describes the results of care institution employees’ self-assessment of their mental health. For the majority, the most stressful factor was the need to work in a health-threatening environment and the sense of responsibility for the residents. Regarding the availability of the forms of support offered to workers experiencing deteriorating mental health due to working in the pandemic situation, more than a third said that their institution did not offer any form of assistance.

**Conclusions: **The article lists a number of recommendations. In the light of the data obtained, it is necessary to increase the intensity of services provided to residents of nursing homes in terms of organizing their free time, meeting their religious and cultural needs and maintaining contact with the social environment.

## Introduction

Nursing homes constitute the largest number ofall care institutions in Poland. These facilities are for people who require round-the-clock care due to age, illness or disability and cannot be provided with the necessary help in the form of community care services. Nursing home residents also suffered the most from the health consequences of the coronavirus disease 2019 (COVID-19) pandemic. By the end of 2020, over a third of nursing home residents (33.9%) had contracted coronavirus ( Statistics Poland, 2021). For comparison, in other 24/7 inpatient facilities, the incidence rate was much lower. For example, 10.9% of all residents of family nursing homes and 4.1% of all residents of homeless shelters contracted the virus ( Statistics Poland, 2021). However, it should be emphasized that the Central Statistical Office, a government administration agency dealing with collecting and sharing statistical data on most areas of public life, does not publish official data on morbidity among medical personnel and care workers. The lack of this type of information significantly limits the ability to objectively assess the effects of the pandemic on the capacity and effectiveness of the care system. Nevertheless, both in Poland and throughout Europe, the evidence-based approach has been a dynamically developing perspective in research on the health of the population and its individual subgroups since the 1990s ( Raine, 1998; Niessen *et al*., 2000).

### Backgrounds

The conducted empirical research was to partially fill the gap in knowledge about the condition of care workers in Poland during the coronavirus pandemic. However, we have consciously gone beyond the narrow analysis of public statistics and taken account of the social aspect of the pandemic. This research is based on the culture-of-disaster perspective (see, among others, Revet, 2020; Revet & Langumier, 2015; Bankoff, 2003; Medina, 2016; Benadusi, 2014), which focuses on ‘what disaster is made of for each of the actors involved’ ( Revet & Langumier, 2015). The subject of interest is the attitudes toward the crisis situation shared by individuals, including how they experienced the reality of the pandemic. Etienne Wenger adds that the relations between the functioning of asocial system and the orientations toward it are shaped within the community of practice ( Wenger, 1998). These practices are revealed not only in action, but also through the shared knowledge, attitudes and beliefs that create a certain common view of the world ( Wenger, 1998, p. 47).

It can therefore be asked why, in the proposed cultural approach, the experiences of care workers should be considered important? Firstly, the knowledge of practitioners should be treated as a social mirror of the resourcefulness of institutions in the crisis caused by the pandemic. The capacity to deliver care services in a pandemic situation may be extended along the continuum between two poles as defined by Nilsson and Olaison: suspension versus seeking new solutions ( Nilsson & Olaison, 2020). Of course, care institutions fulfilled their basic functions despite significant difficulties in operating during the successive waves of the pandemic. However, it is worth referring to the experience of practitioners and asking about the ability to meet the caring, activating, religious and other needs of nursing home residents during the pandemic. This question is answered in the first part of the article, which presents nursing homes employees’ assessments of the individual assistance measures.

Secondly, the analysis of employees’ experiences may become the basis for formulating recommendations on changes in the care system. The previous analyses are dominated by the belief that the pandemic should be treated as a wake-up call ( Fischer *et al*., 2020) in the face of the challenges of an aging society. The academic discourse calls for necessary reforms to ensure high-quality care for people with limited independence in the future ( Daly, 2020). When planning social policies ( Rothman, 2007), it is worth listening to the needs of those who faced the consequences of the pandemic on a daily basis. Based on the analysis of expectations and preferences regarding the protection of mental health, a proposal for changes is presented that would enable better preparation for possible crisis situations caused by the need to work in an emergency.

To sum up, the research carried out in the group of care workers allows the voice of those whose positions in the public discourse are not properly articulated to be captured. On the one hand, they do not have enough time to shape their own narrative in the public discourse as they simply deal with duties related to care and its organization. During the pandemic, this care is even more demanding than before ( Fallon *et al*., 2020; Rodrigues *et al*., 2021). On the other hand, care workers frequently lack representation because they have a ‘silent voice’ due to poorly organized trade unions and employee organizations in comparison with the healthcare sector, among others ( Daly, 2020).

The main objective of this article is to analyze the attitudes of care workers in nursing homes toward the reality of the pandemic in Poland, in four basic contexts: assessment of the degree of social care in the pandemic era; assessment of the most stressful factors since the outbreak of the pandemic; assessment of the mental wellbeing of care workers; and evaluation of institutional measures taken to improve mental health. More specifically, the following hypotheses are tested: 1) the assessment of the degree of social care in the pandemic era differs depending on the type of municipality represented by the respondent (urban, rural, urban-rural), the experience of care workers (measured by seniority), the type of position held (managers or frontline workers) and the type of nursing home (nursing homes for people with mental disorders, nursing homes for people with reduced mobility, nursing homes for the elderly); 2) the assessment of the most stressful factors since the outbreak of the pandemic differs depending on the type of municipality represented by the respondent, the experience of care workers, the type of position held and the type of nursing home; 3) the assessment of the mental wellbeing of care workers differs depending on the type of municipality represented by the respondent, the experience of care workers, the type of position held and the type of nursing home; and 4) the evaluation of institutional measures taken to improve mental health differs depending on the type of municipality represented by the respondent, the experience of care workers, the type of position held and the type of nursing home.

## Methods

### Survey

The field research was carried out in April 2021. It was conducted using the computer-assisted web interviewing (CAWI) technique. The CAWI questionnaire consisted of 19 questions grouped in following topics: mental well-being of employees, internal rules of the functioning of the institution, residents’ needs, cooperation with the environment, birth certificate questions. The questionnaire can be found as *Extended data* ( Necel, 2022). The questionnaire hasn’t been validated.

The CAWI technique was chosen because it provides easy access to the population, which was particularly important during the pandemic due to the need to maintain social distancing. Moreover, this tool allows the avoidance of open-ended questions and questions formulated primarily in the form of statements, making it useful in this project. The link to the questionnaire, along with a covering letter, was sent to the secretariats of nursing homes with a request that two care workers fill in the questionnaire, so that there is no imbalance in the number of questionnaires received between institutions with different numbers of employees. The inclusion criteria (verified by the answers given to the corresponding survey questions) included being engaged in work during the COVID-19 pandemic to April 2021. The exclusion criterion was being an employee who was not a care worker, e.g. an accountant.

### Ethical considerations

The Ethics Committee for Research Involving Human Participants at Adam Mickiewicz University in Poznan granted an approval of the research project **(**Resolution No. 4/2021/2022 adopted on 25 January 2022). Each research participant signed a voluntary and informed consent form for participation in the research. We uploaded “consent form” to a repository.

### Sampling of participants

The research used a non-probability purposive sampling asit was carried out on a relatively small population with a well-known structure with the possibility of accessing all its representatives.

The research was carried out in all nursing homes in five provinces in Poland (there are a total of 16 provinces in Poland): 54 nursing homes in Lower Silesia, 55 in the Lodz Province, 47 in the Subcarpathian Province, 44 in the Lublin Province and 62 in the Greater Poland Province. The provinces were selected for the research based on the analysis of the following variables: the number of nursing homes, the number of places in these homes, the number of residents and the number of employees. On this basis, provinces with similar care resources were selected. The employee population involved: management staff, administration workers and care workers, which included social workers, nurses and therapists, that is, representatives of the profession providing direct support and help to residents of nursing homes.

### Statistical analysis

A chi-square test (with Yates’ correction for 2×2 tables) was used to compare qualitative variables among the groups. In the case of low values in the contingency tables, Fisher’s exact test was used instead. The level of significance for all statistical tests was set at 0.05. Thus, all p values below 0.05 were interpreted as showing significant correlations. R 4.0.5 was used for the computations (RRID:SCR_001905).

## Results

### The studied group of care workers

By the end of 2020, 37,309 care workers were employed in Poland. In the five voivodships surveyed, a total of 18,659 carers were employed. The research was conducted among 189 of them ( Ministry of Family and Social Policy, 2022). Employees representing three types of care facilities participated in the research: nursing homes for people with mental disorders – 36.5%, nursing homes for people with reduced mobility – 36.5%, and nursing homes for the elderly – 27.0%. The nursing homes whose employees took part in the research were located primarily in urban municipalities – 56%, followed by urban-rural municipalities – 19.8%, and rural municipalities – 24.2% ( Necel, 2022).

Respondents were most often care workers – 44.9% (of whom 18.6% were nurses) and slightly less often management staff – 29.9%, while 21.6% of all respondents were administration workers. As a rule, respondents had extensive work experience in the social welfare sector. Over one-third (36.2%) had more than 20 years of work experience. There was also a large group of employees with 10 to 19 years of work experience, who constituted 33.3% of all respondents. Only 11.9% of respondents had less than two years of work experience, and 18.6% of the surveyed nursing home employees had been employed for two to nine years.

### Assessment of the services provided in nursing homes

Respondents were asked to assess to what extent the basic needs of residents were met during the pandemic ( Table 1). The question was accompanied by multiple-choice answers detailing the various activities of care institutions in this area. Most respondents assessed the fulfillment of the basic living needs of residents (food, clothing, hygiene products) as definitely good (78.3%) and somewhat good (20.6%). Similarly, the represented institutions were rated highly in terms of the availability of care and support services. The implementation of this function was assessed as definitely good by 64.6% of respondents, or as somewhat good by 32.8%. In other aspects of the functioning of the nursing homes, respondents had more diverse opinions. When it comes to organizing the leisure time of residents, 24.3% assessed it as definitely good, and 46.6% as somewhat good. On the other hand, 14.3% of respondents were of the opposite opinion and answered ‘somewhat bad’ or ‘very bad’. Just over half of respondents (54.5%) assessed the fulfillment of religious and cultural needs as definitely good or somewhat good, and about one-fifth of respondents had the opposite opinion (22.8% – the cumulative percentage of answers ‘somewhat bad’ and ‘very bad’). In the context of the restrictions introduced on visits by outsiders during the pandemic, it is surprising that 21.2% of respondents assessed activities related to maintaining and developing contact with family and the social environment as definitely good and as many as 42.9% as somewhat good. It is worth emphasizing, however, that over one-fifth of respondents had a different opinion (21.2% – the cumulative percentage of answers ‘somewhat bad’ and ‘very bad’), and 14.8% of employees did not have an opinion on this subject. The activities of care institutions for the benefit of the local community were rated the least positively, as 5.3% answered ‘definitely good’, 18% ‘somewhat good’, and as many as 55.6% of respondents did not express an opinion on this subject.

**Table 1.** Assessment of the services provided (N = 189).

Type of services provided by a nursing home	Assessment				
	Definitely bad	Somewhat bad	I have no opinion	Somewhat good	Definitely good
Satisfying the living needs of residents	0 (0.00%)	0 (0.00%)	2 (1.06%)	39 (20.63%)	148 (78.31%)
Providing care and support services	0 (0.00%)	0 (0.00%)	5 (2.65%)	62 (32.80%)	122 (64.55%)
Organizing residents’ free time	3 (1.59%)	24 (12.70%)	28 (14.81%)	88 (46.56%)	46 (24.34%)
Fulfilling religious and cultural needs	10 (5.29%)	33 (17.46%)	43 (22.75%)	74 (39.15%)	29 (15.34%)
Maintaining and developing contact with family and the social environment	8 (4.23%)	32 (16.93%)	28 (14.81%)	81 (42.86%)	40 (21.16%)
Taking actions for the benefit of the local community	17 (8.99%)	23 (12.17%)	105 (55.56%)	34 (17.99%)	10 (5.29%)

The statistical analyses conducted (chi-square test or Fisher’s exact test) showed that the assessments of the functioning of nursing homes during the pandemic were different depending on the respondents’ job position. Significant differences were noted in the opinions regarding the fulfillment of religious and cultural needs (p < 0.001, probability value < 0.05), the implementation of which was assessed as definitely good (28.4%) and somewhat good (33.3%) by care workers, while only 2% of directors expressed an overwhelmingly positive opinion, and 38%a somewhat positive opinion about this aspect. The job position also differentiated the views on maintaining contact with the family and the social environment (p = 0.034, probability value < 0.05). In this case, care workers were also more optimistic in their assessments. This aspect was assessed as definitely good by 29.6%, or as somewhat good by 39.5%. Only 10% of management representatives gave a similar opinion, responding ‘definitely good’, and 40% responded ‘somewhat good’. The job position was also important when assessing activities undertaken for the benefit of the local environment (p = 0.008, probability value < 0.05). As was the case with the above dependencies, this time care workers were also more satisfied than the management of the facility. While 8.6% of care workers assessed this function as definitely good and 17.2% as somewhat good, only 2% of the management staff assessed it as definitely good and14% as somewhat good.

In the course of the analyses, the hypothesis has been confirmed that the type of nursing home in which an employee works significantly differentiates the assessment of the functioning of the care institution in the context of meeting the basic needs of residents. A statistically significant relationship was noted in the area of providing care and support services (p = 0.009, probability value < 0.05). Namely, 73.9% of employees of nursing homes for people with mental disorders assessed this aspect of activity as definitely good. In the case of nursing homes for the elderly, 66.6% of answers were definitely good, while in the case of nursing homes for people with reduced mobility, this number was 53.6%. The differentiation also concerned the organization of leisure time for residents (p = 0.002, probability value < 0.05). Namely, 37.6% of respondents from nursing homes for people with mental disorders expressed a definitely good opinion about this form of support. Only 23.5% of the representatives of nursing homes for the elderly and 11.5% of respondents from nursing homes for people with reduced mobility were of a similar opinion. Significant differences were also noted in terms of maintaining and developing contact with family and the environment (p = 0.046, probability value < 0.05). They were assessed the best by the representatives of nursing homes for people with mental disorders (27.5% definitely good, and the worst by respondents from homes for people with reduced mobility (13% definitely good). The analyses of statistical dependencies did not show that the type of municipality or the respondent’s seniority statistically significantly differentiated the assessment of the functioning of a care institution.

### The most stressful factors at work during the pandemic

Respondents were asked about the most stressful factors at work since the declaration of the pandemic ( Table 2). The surveyed nursing home employees most often indicated the necessity to work under the conditions of health risk as a stressful factor. This answer was given by 68.3% of respondents.

**Table 2.** The most stressful factors at work during the coronavirus disease 2019 (COVID-19) pandemic (N = 189).

The main stressors	n	%
Working in hazardous health conditions	129	68.25%
Responsibility for the residents of the nursing home	118	62.43%
Combining work with duties toward loved ones	67	35.45%
Unclear procedures at the national level	62	32.80%
Responsibility for other people in private life	46	24.34%
Limited possibility of testing for the presence of coronavirus	36	19.05%
Conflicts arising due to tension in the workplace	34	17.99%
Unclear procedures at the local level	18	9.52%
Lack of/too little personal protective equipment (PPE) in the workplace	8	4.23%
Unclear procedures at the institution level	8	4.23%
No other resources	5	2.65%
Conflicts arising due to tension in private life	4	2.12%
Other	6	3.17%

Note. The percentages do not add up to 100 as it was a multiple-choice question.

Another stressful factor for respondents was their responsibility for the residents of nursing homes, which was emphasized by 62.4%. Other stressors were mentioned less frequently. More than one-third of respondents (35.4%) mentioned combining work with duties toward their relatives, such as taking care of children and other dependent family members. In the light of the empirical data obtained, unclear procedures in the pandemic situation also turned out to be stressful at three levels: national, local and at the level of the institution where respondents worked. Nearly one-third of respondents pointed to the lack of clear procedures at the national level (32.8%). Interestingly, the lack of clear procedures at the local level was indicated as a stressful factor by 9.5% of respondents, while unclear procedures inside an institution were pointed out by 4.2%.

Responsibility for others outside the workplace was a stressful factor for almost every fourth respondent (24.3%). However, for every fifth respondent (19%), stress and tension resulted from the limited possibility of testing for the presence of coronavirus. The lack of or too little personal protective equipment (PPE) in the workplace was selected as a stressor by only 4.2% of respondents.

Conflicts emerging due to tension in the workplace were stressful for 18% of respondents, while in their private life it was 2.1% and thus the least frequently chosen stress factor.

The analyses conducted (chi-square test and Fisher’s exact test) have confirmed the hypothesis that opinions about stressors differ depending on seniority. Statistically significant differences were noted in the opinions on combining work with responsibilities toward loved ones (p = 0.005, probability value < 0.05). This issue was mentioned by 57.5% of respondents with work experience from three to nine years, and only by 19% of those with work experience of up to two years. Seniority significantly differentiated the employees in their statements about unclear procedures at the local level (p = 0.026, probability value < 0.05), which was a source of stress mainly for respondents with up to two years of experience, among whom 28.5% pointed to this problem. However, this factor was stressful for only 9.3% of people with the longest work experience (20 years and more). The analyses have also confirmed the hypothesis about the relationship between the position held and the importance given to unclear procedures at the local level (p = 0.029, probability value < 0.05). This problem was much more often emphasized as stressful by administration employees (11.1%). None of the management staff representatives indicated the importance of this factor. However, the sense of responsibility for the residents (p < 0.001, probability value < 0.05) was much more likely to be a stressor for institution directors (88%) than for administration employees (47.2%) or care workers (56.7%).

There were no statistically significant correlations confirming the hypothesis about differences in the perception of stressors depending on the type of the municipality represented or the type of nursing home.

### Assessment of mental health

The question about stress factors at work corresponds to another research problem concerning the mental health of nursing home workers during the pandemic ( Table 3). Respondents were asked a series of questions based on the following template ‘Did you … in the months since the outbreak of the pandemic?’ with selected areas of wellbeing clarified. They were asked to respond based on the following multiple-choice answers: ‘much more than usual’, ‘slightly more than usual’, ‘the same as usual’, ‘slightly less than usual’ and ‘much less than usual’. Areas of particular interest were those that changed during the pandemic and so the cumulative percentages of the responses ‘much more than usual’ and ‘slightly more than usual’ are presented to describe the results.

**Table 3.** Self-assessment of mental health during the coronavirus disease 2019 (COVID-19) pandemic (N = 189).

Mental health	Assessment				
	Much less than usual	Slightly less than usual	The same as usual	Slightly more than usual	Much more than usual
Stress	2 (1.06%)	2 (1.06%)	26 (13.76%)	74 (39.15%)	85 (44.97%)
Fatigue	1 (0.53%)	2 (1.06%)	31 (16.40%)	92 (48.68%)	63 (33.33%)
Sleep problems	2 (1.06%)	4 (2.12%)	81 (42.86%)	64 (33.86%)	38 (20.11%)
Nervousness in dealing with others	3 (1.59%)	1 (0.53%)	70 (37.04%)	90 (47.62%)	25 (13.23%)
Anxiety, severe anxiety	2 (1.06%)	1 (0.53%)	54 (28.57%)	90 (47.62%)	42 (22.22%)
Mobilization to act	0 (0.00%)	13 (6.88%)	40 (21.16%)	60 (31.75%)	76 (40.21%)
Feeling no pleasure	7 (3.70%)	12 (6.35%)	106 (56.08%)	41 (21.69%)	23 (12.17%)
Perceiving the situation as hopeless	5 (2.65%)	9 (4.76%)	81 (42.86%)	63 (33.33%)	31 (16.40%)

The research results indicate the widespread experience of increased stress among nursing home employees. Over 84% of respondents felt more stressed than usual on a daily basis. The vast majority of respondents (over 70%) felt motivated to act at that time. Most respondents also experienced chronic fatigue (82%), and more than half had trouble sleeping (54%).

The intensity of neurotic reactions was also greater than usual. Almost 70% of research participants felt more anxiety or severe anxiety than usual. Over 60% of respondents felt increased nervousness and tension in contacts with people.

The studied individuals relatively less frequently manifested selected depressive reactions in the form of cognitive changes consisting of perceiving the situation as hopeless (less than 50%) and the inability to experience pleasure (slightly over 33%).

The hypothesis that psychological wellbeing is related to the position held has been confirmed by the analysis of statistical correlations in only one aspect, that is, the mobilization to act (p < 0.001, probability value < 0.05). It was felt most strongly by directors, as many as 68% of whom felt it much more than usual, and the weakest by administrative employees, only 25% of whom were mobilized to act much more than usual. It turns out that the differences in the mobilization to act were also influenced by the type of nursing home in which a respondent works (p = 0.013, probability value < 0.05). The mobilization function of the pandemic was higher in facilities for the elderly, where 45.1% of respondents felt this much more than before the pandemic, while in facilities for people with reduced mobility and mental disorders, it was 36.2% and 40.5%, respectively. The hypothesis that the type of municipality represented differentiates the psychological wellbeing of respondents has also been confirmed. Statistically significant differences were noted in the case of nervousness in contacts with others (p = 0.015, probability value < 0.05), which was felt much more than usual by nursing home employees from urban-rural municipalities (27.7%) than by those from rural (13.6%) or urban (7.8%) ones.

### Protection of mental health: available and expected forms of support for care workers

In the research, respondents were asked about solutions used in their workplaces to protect mental health ( Table 4). For this purpose, respondents were asked the following closed-ended question: ‘What solutions have been implemented to protect the mental health of people working in your institution?’ The following forms of support were distinguished among the responses: ‘conversation with a psychologist’, ‘support group’, ‘supervision’ and ‘other’. Over one-third of respondents (37%) did not record any activities in their institution aimed at protecting mental health during the pandemic. This means they did not choose any of the possible answers. Among 63% of respondents who thought that their institution had implemented support instruments, most mentioned a conversation with a psychologist. Such a solution was observed by over one-third (35.4%) within their institution. According to some respondents, the institutions also organized support groups. This type of assistance was indicated by approximately one-fifth of respondents (21.7%). Moreover, 7.4% of respondents could have performed their work under supervision, which can also be classified as a solution aimed at protecting mental health. Finally, 11.1% of respondents indicated other types of support.

**Table 4.** Available forms of psychological support (N = 189).

Psychological support	n	%
No support whatsoever	70	37.04%
Conversation with a psychologist	67	35.45%
Support group	41	21.69%
Supervision	14	7.41%
Other	21	11.11%

The analysis of statistical correlations performed using the chi-square test did not show significant differences in the opinions on the forms of psychological support provided depending on the position held, seniority, the type of nursing home and the type of municipality in which the facility was situated.

Respondents were also asked the following question about their expectations toward the preferred forms of support for mental wellbeing: ‘What kind of activities aimed at protecting the mental health of employees would you expect in your institution?’ Respondents had the opportunity to express their own expectations toward the represented institution. The empirical material collected was categorized into several responses.

Most respondents (23.8%) expected a conversation with a psychologist. Several times respondents indicated that a psychologist should be from outside the institution, which may result from the reluctance to reveal one’s thoughts to a person one knows due to the lack of anonymity. Support groups were another most frequently mentioned activity aimed at protecting the mental health of employees that, according to the surveyed persons, should have been organized in their institution (11.1%). Another category of answers related to rest or holidays– this was indicated by 6.3% of respondents. Every twentieth respondent (5.2%) indicated the need for the institution to organize training sessions and workshops to help them cope better in the pandemic situation. The same percentage of respondents indicated supervision (5.2%) as well as the need to employ additional people (4.8%). Other responses included expectations related to remuneration (higher salary, bonuses), the role of management (‘clearer decisions of the managers’, ‘support from the managers’) as well as the bodies running the institutions (‘interest of the unit in charge’). There were also references to the situation outside the institutions. Respondents indicated that the following would be helpful: ‘clear procedures at the national level’ or a change in the climate of public opinion, which one of the respondents expressed as: ‘the end of the state of the media pandemic panic’.

## Discussion

The provision of care requires direct interpersonal contacts and spatial closeness ( Weicht, 2015). Maintaining these conditions is a particularly difficult task in the era of the COVID-19 pandemic, where care workers help others and risk their own health ( Devlieghere & Roose, 2020) and often their life, as indicated in some countries by higher mortality rates among care workers than in the entire population ( Daly, 2020). In medical care, from the beginning, there was general agreement in the medical community to calculate the benefits of providing procedures that do not directly save life and health relative to the ‘costs of contact’, that is, the possibility of contracting the virus in contacts with the healthcare system ( Bhatia *et al*., 2021). The costs of direct contact were also calculated in social care, as evidenced by the empirical data obtained. On the one hand, ensuring social activation of residents, concern for their spiritual development or integration with the local environment was associated with a higher risk of infection understood as a cost in this context, which could directly translate into a lower assessment of these forms of support. On the other hand, regardless of this cost, the implementation of basic rights, such as subsistence rights and security rights ( Shue, 1980), can be considered satisfactory. This is evidenced by the high assessment of the fulfillment of living and care needs obtained regardless of the prevailing pandemic conditions. However, the employees of care institutions were relatively less satisfied with the implementation of freedom rights relating to the need for individual autonomy ( Fabre, 2004). Consequently, activities in the areas of social activation, spiritual development and integration with the local environment, which required contacts with people and entities from outside the institutions, were assessed as less satisfactory. These could have been associated with a greater risk of infection, and the cost of care could have been treated as too high relative to profit.

In the context of questions about providing assistance to residents, despite the above-mentioned differences in assessments, it is worth emphasizing that positive assessments outweighed negative ones in each dimension of assistance activities. A particularly high assessment of the fulfillment of the living and care needs was possible thanks to the great commitment of care workers. Not without significance was the amendment to the Act on the posting of employees as part of the provision of services introduced in 2020, giving nursing home managers the ability to ask their employees to provide the necessary overtime work after obtaining their prior consent ( Amendment of the act on the posting of workers as part of the provision of services and some other acts, 2020). This administrative decision made it possible to increase the number of care workers, who were seriously threatened by COVID-19 around the world ( Comas-Herrera *et al*., 2020), including in Poland. It is worth highlighting that there were relatively fewer positive assessments when it comes to providing religious services to residents ( Swift, 2020; Drummond & Carey, 2020). Pastoral care is of great importance for believers, especially when they experience suffering and illness ( Swift, 2020), and spiritual support can improve wellbeing and physical condition (Koening, 2020). Therefore, cooperation with churches and religious associations is significant.

At the peaks of the successive waves of the coronavirus in Poland, which were in March and April 2020, October and November 2020, and in March and April 2021, nursing homes were closed to visitors or contact with outsiders was significantly limited. In this context, it is surprising that the activities aimed at maintaining and developing contact between residents and their families and relatives were highly rated. This is because during the lockdown, the institutions’ employees tried to keep in touch with residents’ relatives using new teleinformation technologies (mainly instant messaging). Despite the many risks and weaknesses associated with the use of new technologies: insufficient digital competences of users ( de Jonge *et al*., 2020), a lack of trust in technology and discomfort in its use –primarily among seniors ( Swinford *et al*., 2020), p. 519), it is likely that new communication technologies will stay in social work and care for longer ( Scheyett, 2020). Therefore, it is worth considering how to permanently include them in the mainstream of care and how to use them to build effective relationships with residents’ relatives. The assessments of available forms of support presented in this article only took into account the perspective of care workers. Cognitively, it would be interesting to continue the research among the residents themselves using qualitative methods, such as individual in-depth interviews, to capture the experiences of people who require care and have to face the constraints imposed due to the pandemic.

Working in a situation where there is a high risk of losing health translates into weakened mental health ( Khatri *et al*., 2019). This observation was also confirmed in the conducted research. Nursing home workers noted significant personal and emotional labor costs during the pandemic. The sense of responsibility for others, that is, residents of the nursing home and their own family members, was the dominant stress factor for them. The literature raises the issue of a specific conflict that may occur between the need to protect oneself and one’s loved ones, and the sense of responsibility in the context of fulfilling the professional role of a care worker ( Baum, 2012; Dekel & Baum, 2010). Additionally, working in a nursing home during the pandemic is an exemplification of the idea of ‘shared traumatic reality’ which consists of being subject to the same consequences of a crisis situation as the client ( Cain, 2015). Consequently, this can lead to blurring boundaries between a worker and a client, as well as to greater emotional distress (Caroll *et al*., 2010).

Under conditions of stress and weakened mental health, activities supporting employees in coping with this type of problem are of great importance. On the one hand, administrative measures should be taken at the management level of a specific institution to increase work safety, such as increasing the availability of PPE, changing the organization of work, including delegating employees to other activities, or increasing the number of working hours. In this area, it is also important to create appropriate financial incentives ( Garbers & Konradt, 2014; Landry *et al*., 2017) or compensation for work in extraordinary conditions. On the other hand, it is also necessary to take care of the emotional wellbeing of employees by providing them with appropriate support in the form of professional self-care ( Skinner, 2015). In social work, the importance of professional self-care in counteracting distress and supporting a healthier, sustainable workforce is emphasized (Bloomquist, 2015). Hence the need to educate students of welfare professions in professional self-care ( Newell & Nelson-Gardell, 2014) and to organize professional support of supervisors and psychologists for experienced care workers.

This research has several limitations. Firstly, the research sample included nursing home employees from five provinces in Poland. Therefore, care should be taken not to generalize (extrapolate) the research results to all care workers throughout the country. Secondly, the research was carried out using the CAWI technique, which was the only possible way to safely conduct the research during the pandemic. However, it limits the possibility of verifying whether a respondent was a nursing home employee actually working during the pandemic. Thirdly, the opinions expressed were undoubtedly influenced to some extent by the context in which the surveys were carried out. This was when the respondents were experiencing the third wave of the pandemic. On the one hand, they were not sure how long the crisis situation would last and what its far-reaching consequences would be for the functioning of the institution and its clients. On the other hand, they already had a certain sense of security guaranteed by vaccinations provided to all care workers and residents.

## Conclusion

In this research, stressors have been identified and changes in the mental wellbeing of nursing home workers during the pandemic have been described. An in-depth analysis of this data makes it possible to design and implement forms of support most suitable for the reported needs, based on the direct experiences of care workers. The organization of various forms of mental health support for formal care workers, including nursing staff, is the main challenge facing the social welfare system in Poland. The offer should include individual psychological help (provided by a psychologist from outside the institution), supervision and peer support groups. It is also necessary to monitor the effectiveness of activities aimed at protecting the mental health of employees (continuous evaluation) and introduce possible modifications in order to increase their effectiveness. In the light of the data obtained, it is also necessary to increase the intensity of services provided to residents of nursing homes in terms of organizing their free time, meeting their religious and cultural needs and maintaining contact with the social environment. In this context, it is necessary to develop innovative (in Poland) forms of support, such as laughter therapy ( Kuru & Kublay, 2017) or meditation-relaxation strategies ( Lindberg, 2005), which not only support spiritual growth and reduce negative emotional states, but are also an interesting way of spending free time. It is worth noting that this type of activity can be conducted remotely using video communication apps.

## Data availability

### Underlying data

Open Science Framework: The Coronavirus Pandemic in Nursing Homes – the Experience of Care Workers in Poland, https://doi.org/ 10.17605/OSF.IO/72NM4 ( Necel, 2022).

### Extended data

Open Science Framework: The Coronavirus Pandemic in Nursing Homes – the Experience of Care Workers in Poland, https://doi.org/ 10.17605/OSF.IO/72NM4 ( Necel, 2022).

This project contains the following extended data:

• Questionnaire
• Cover letter
• Consent form

Data are available under the terms of the Creative Commons Zero “No rights reserved” data waiver (CC0 1.0 Public domain dedication).
