# Supplementary material for: The coronavirus disease 2019 (COVID-19) pandemic in nursing homes – the experience of care workers in Poland
Source: F1000Res. 2023 Mar 22;11:1008. Originally published 2022 Sep 7. [Version 3] doi: 10.12688/f1000research.124984.3 (PMC9999047; doi:10.12688/f1000research.124984.3)
Supplement: Peer review history

# Peer review: Reviewer response for version 2

**DOI:** 10.5256/f1000research.143438.r163423

**Published:** 2023-03-09

**Authors:** Florin Lazar

**Affiliations:** 1 School of Sociology and Social Work, University of Bucharest, Bucharest, Romania

I have read the revised manuscript and I still have some minor editing to suggest:

- The first comment regarding the first sentence about the typo "ofall" to be changed to "of all" was not corrected.
- The titles of references from Statistics Poland were not translated into English, as requested.
- In the new text (Discussion section), I suggest to replace the word "orphanages" which is stigmatising with more neutral ones "residential institutions" or "residential care" .

Is the work clearly and accurately presented and does it cite the current literature?

Partly

If applicable, is the statistical analysis and its interpretation appropriate?

Partly

Are all the source data underlying the results available to ensure full reproducibility?

Yes

Is the study design appropriate and is the work technically sound?

Partly

Are the conclusions drawn adequately supported by the results?

Partly

Are sufficient details of methods and analysis provided to allow replication by others?

Yes

Reviewer Expertise:

NA

I confirm that I have read this submission and believe that I have an appropriate level of expertise to confirm that it is of an acceptable scientific standard.

# Response

**Published:** 2023-03-20

**Authors:** Ryszard Necel

**Affiliations:** Adam Mickiewicz University, Poland

Dear Professor, 

 I wanted to thank you for your review. I have made the suggested changes to the text. 

 Kind regards, 

 Ryszard Necel

# Peer review: Reviewer response for version 1

**DOI:** 10.5256/f1000research.137239.r154261

**Published:** 2023-01-03

**Authors:** Florin Lazar

**Affiliations:** 1 School of Sociology and Social Work, University of Bucharest, Bucharest, Romania

The manuscript is generally well structured and written. The main topic is relevant for policy-making and brings into the debate about the impact of the Covid-19 pandemic the perspective of care workers in nursing homes. The experiences from the Polish respondents could be relevant to other contexts as well, but the manuscript needs some revisions before it is approved.

 Here are some comments and suggestions for improvements:

 The first sentence needs a ‘space’ in the word ‘ofall’ to read “Nursing homes constitute the largest number of all care institutions in Poland. ”

 The reference to Statistics Poland need to be translated into English also (in the Reference section).

 Is not clear why the author consider that data on morbidity of medical and care staff during the pandemic explains the impact of the pandemic on the care system. Also, the phrasing ‘capacity and effectiveness of the care system’ is not clear enough as it does not explain the type of capacity (capacity to do what?), nor what is meant by effectiveness of the care system (presumably to protect the service users from the negative consequences of the pandemic - from this point of view, maybe data on morbidity of residents could be more relevant). The next idea is not linked to the previous phrase, as the expansion of evidence-based health services does not explain the insufficient data about the care system in Poland. Moreover the argument is that evidence-based research developed over the past 30 years on the ‘health of the population’, but the focus of the current research is on the perceptions of care workers and less on their health (apart from mental health). The title is a bit misleading as the research is focused on care workers in nursing homes and not all care workers (e.g. in community-based services, foster care, etc.).

 Change ‘Backgrounds’ to ‘Background’.

 The ‘culture-of-disaster’ approach is not referred back in the Discussion section, but only presented in the Introductory section. It would have been relevant to discuss to what extent the data collected confirmed the initial theoretical perspective.

 The Background section is missing information about the situation of nursing homes in Poland and why were these institutions were chosen. Some information is provided later on, but I suggest to move the text somewhere in the Background section.

 The objective and hypotheses are not clear enough, as is stated that assessments are made, but is not sufficiently explained how these assessments are made. These are rather perceptions or attitudes, not really assessments. For instance ‘assessment of the degree of social care’ is not clear, nor it is operationalised to understand what is meant my degree of social care, so I would suggest to avoid using the terms ‘assessments’ and  ‘hypotheses’, but rather ‘attitudes’ or ‘perceptions’ and ‘research questions’. The alternative is to provide sufficient details about how the assessment and the concepts from the hypotheses are measured. A quantitative research does not always include hypotheses, if it is descriptive (see Rubin, A., & Babbie, E. R. (2016). Empowerment series: Research methods for social work. Cengage Learning.).

 The urban-rural option for residence is unusual and the author need to explain how a place is both urban and rural?

 The current hypotheses are not clear-cut, being too general and comprising too many variables to test. If kept, I suggest to choose a more precise statement - e.g. managers are more likely to be worried about the general impact of the pandemic on the institution than frontline workers. Then every concept is detailed on how it is measured. Currently no information is available on how the concepts are measured - e.g. degree of social care, stressful factors, mental wellbeing, institutional measures. The reader find out only in the Results section something about these. Irrespective if the author decides to keep the hypotheses or not, these changes are needed.

 The survey is presented as an interview (CAWI), so I suggest to adjust the text to reflect the situation. From the questionnaire presented as Extended data, it seems that Microsoft Forms from the software Microsoft 365 package was used.

 Regarding Ethical approval, it seems that it was obtained after data collection and not before as it usually is needed. However, the data collection process did not pose ethical concerns, as participation was voluntarily.

 The reference to ‘(Ministry of Family and Social Policy, 2022)’ from the second sentence of the Results section need to be moved after the first sentence and not after the second sentence, since it gives information about the target population and not about the number of those included in the research. However, some info about the coverage of the study participants would be necessary. In the five provinces included in the research there were about 262 nursing homes (54 nursing homes in Lower Silesia, 55 in the Lodz Province, 47 in the Subcarpathian Province, 44 in the Lublin Province and 62 in the Greater Poland Province). Since the number of respondents was 189, we can assume there were responses from about 95 institutions, out of 262 (about 36% coverage rate; another option is to report the coverage rate based on the total number of all employees in nursing homes in the five provinces - about 1%).

 I suggest to add a table describing the study population in the Results section.

 Regarding the presentation of the results, I suggest to present the tables with the correlations (including significance coefficients) instead of the frequencies. Also, I suggest to either group negative vs. positive responses, or calculate means and present the results referring to the hypotheses, if kept.

 In the sub-section ‘Assessment of mental health’ the whole first paragraph could be moved into a new sub-section within Methods section, ‘Measures’ and add info about the other measures used (e.g. in the next sub-section, the first 4 sentences + the paragraph about the expected support could be also moved).

 The Discussion section could start with a summary of the main findings and then point out when is referring to own findings and when to those arising from the literature.

 I suggest to add a limitation of the research related to the conflict of interest of respondents assessing their work, presumably being more likely to be positive.

 The suggestion to provide ‘laughter therapy (Kuru & Kublay, 2017) or meditation-relaxation strategies (Lindberg, 2005),’ does not derive from the research itself, so I suggest to remove it or to add something more general, as I assume there are other forms of support available. I also suggest to end with a more general conclusion.

Is the work clearly and accurately presented and does it cite the current literature?

Partly

If applicable, is the statistical analysis and its interpretation appropriate?

Partly

Are all the source data underlying the results available to ensure full reproducibility?

Yes

Is the study design appropriate and is the work technically sound?

Partly

Are the conclusions drawn adequately supported by the results?

Partly

Are sufficient details of methods and analysis provided to allow replication by others?

Yes

Reviewer Expertise:

Social work; Social policy

I confirm that I have read this submission and believe that I have an appropriate level of expertise to confirm that it is of an acceptable scientific standard, however I have significant reservations, as outlined above.

## References

[1] : Empowerment series: Research methods for social work. *Cengage Learning* .2016;

# Response

**Published:** 2023-01-19

**Authors:** Ryszard Necel

**Affiliations:** Adam Mickiewicz University, Poland

The first sentence needs a ‘space’ in the word ‘of all’ to read “Nursing homes constitute the largest number of all care institutions in Poland.

**The sentence is corrected  - p. 2**

 The reference to Statistics Poland need to be translated into English also (in the Reference section).

**The reference is corrected  - p. 21**

 Is not clear why the author consider that data on morbidity of medical and care staff during the pandemic explains the impact of the pandemic on the care system.

**The text has been added - p. 2-3**

 the argument is that evidence-based research developed over the past 30 years on the ‘health of the population’, but the focus of the current research is on the perceptions of care workers and less on their health (apart from mental health)

**The sentence is corrected  - p. 3**

 The title is a bit misleading as the research is focused on care workers in nursing homes and not all care workers (e.g. in community-based services, foster care, etc.).

**In the first sentence of the title it indicates nursing homes**

 Change ‘Backgrounds’ to ‘Background’.

**The sentence is corrected  - p. 3**

 The ‘culture-of-disaster’ approach is not referred back in the Discussion section, but only presented in the Introductory section. It would have been relevant to discuss to what extent the data collected confirmed the initial theoretical perspective.

**The text has been added – p. 15**

 The Background section is missing information about the situation of nursing homes in Poland and why were these institutions were chosen. Some information is provided later on, but I suggest to move the text somewhere in the Background section.

**The text has been added – p. 3**

 The objective and hypotheses are not clear enough, as is stated that assessments are made, but is not sufficiently explained how these assessments are made. These are rather perceptions or attitudes, not really assessments. For instance ‘assessment of the degree of social care’ is not clear, nor it is operationalised to understand what is meant my degree of social care, so I would suggest to avoid using the terms ‘assessments’ and  ‘hypotheses’, but rather ‘attitudes’ or ‘perceptions’ and ‘research questions’. The alternative is to provide sufficient details about how the assessment and the concepts from the hypotheses are measured. A quantitative research does not always include hypotheses, if it is descriptive (see Rubin, A., & Babbie, E. R. (2016). Empowerment series: Research methods for social work. Cengage Learning.).

**1) As suggested by the second reviewer. I removed hypotheses and replaced them with research questions  - p. 3**

**I have seen Rubin, A., & Babbie, E. R. (2016). Empowerment series: Research methods for social work. Cengage Learning. Thank you for this book.**

**2) The section “Description of variables” has been added. I have presented in the table type of variables and items. Now I hope is clearer what is meant the main variables – p.7-8**

**3) I also removed the term “assessment” and replaced it with “perception”.** 

 Regarding the presentation of the results, I suggest to present the tables with the correlations (including significance coefficients) instead of the frequencies. Also, I suggest to either group negative vs. positive responses, or calculate means and present the results referring to the hypotheses, if kept.

**The analysis of each variable was separately (seniority, type of commune, etc.). Then it would be necessary to present several tables in a short manuscript.**

 some info about the coverage of the study participants would be necessary.

**The text has been added – p. 8**

 I suggest to add a table describing the study population in the Results section.

**The table has been added – p. 8-9**

 The urban-rural option for residence is unusual and the author need to explain how a place is both urban and rural?

**The text has been added – p. 3**

 The Discussion section could start with a summary of the main findings and then point out when is referring to own findings and when to those arising from the literature.

**The text has been added – p. 15**

 I suggest to add a limitation of the research related to the conflict of interest of respondents assessing their work, presumably being more likely to be positive.

**The text has been added – p. 17**

 The suggestion to provide ‘laughter therapy (Kuru & Kublay, 2017) or meditation-relaxation strategies (Lindberg, 2005),’ does not derive from the research itself, so I suggest to remove it or to add something more general, as I assume there are other forms of support available. I also suggest to end with a more general conclusion

**The text has been removed**

 I also suggest to end with a more general conclusion

**The text has been added – p. 18**

# Peer review: Reviewer response for version 1

**DOI:** 10.5256/f1000research.137239.r149759

**Published:** 2022-10-25

**Authors:** Ewa Kantowicz

**Affiliations:** 1 Department of Social Pedagogy and Educational Research Methodology, University of Warmia and Mazury in Olsztyn, Olsztyn, Poland

**1. Evaluation of the research background**

 The conducted empirical research was to partially fill the gap in knowledge about the condition of care workers in Poland during the coronavirus pandemic. The research was based on the culture-of-disaster perspective presented e.g. by Sandrine Revetand and Julien Laugumier, which focuses on ‘what disaster is made of for each of the actors involved’ ( Revet & Langumier, 2015).

 The subject of interest was focused on the attitudes toward the crisis situation shared by individuals, including how they experienced the reality of the pandemic. The proposed cultural approach, the experiences of care workers were considered as the important ones, because they were treated as a social mirror of the resourcefulness of institutions in the crisis caused by the pandemic. The issues raised undoubtedly determine the importance of the issue raised in the context of resident care for dependent persons, but also the use of knowledge and experience from the pandemic period of employees of these institutions in securing adequate medical, psychological and social care and a support system corresponding to the needs of the wards.

**2. Evaluation of methodology**

 The field research was carried out in April 2021. It was conducted using the computer-assisted web interviewing (CAWI) technique. The CAWI questionnaire consisted of 19 questions grouped in following topics: mental well-being of employees, internal rules of the functioning of the institution, residents’ needs, cooperation with the environment, birth certificate questions. The CAWI technique was chosen because it provided easy access to the population, which was particularly important during the pandemic due to the need to maintain social distancing. This tool allowed also the avoidance of open-ended questions and questions formulated primarily in the form of statements, making it useful in this project. The statistical analyses conducted (chi-square test or Fisher’s exact test) showed that the assessments of the functioning of nursing homes during the pandemic were different depending on the respondents’ job position.

 In the course of the analyses, the hypothesis has been confirmed that the type of nursing home in which an employee works significantly differentiates the assessment of the functioning of the care institution in the context of meeting the basic needs of residents. It was interesting that the surveyed nursing home employees most often indicated the necessity to work under the conditions of health risk as a stressful factor. Another stressful factor for respondents was their responsibility for the residents of nursing homes. The analyses conducted (chi-square test and Fisher’s exact test) had confirmed the hypothesis that opinions about stressors differ depending on seniority. Statistically significant differences were noted in the opinions on combining work with responsibilities toward loved ones. The analyses also confirmed the hypothesis about the relationship between the position held and the importance given to unclear procedures at the local level. The research results indicated the widespread experience of increased stress among nursing home employees. The analysis of statistical correlations performed using the chi-square test did not show significant differences in the opinions on the forms of psychological support provided depending on the position held, seniority, the type of nursing home and the type of municipality in which the facility was situated. In the research, stressors had been identified and changes in the mental wellbeing of nursing home workers during the pandemic period.

 The research methodology was used correctly and allowed to formulate some proposals for social practice. The analysis of the data allowed to design and implement forms of support most suitable for the reported needs, based on the direct experiences of care workers. The organization of various forms of mental health support for formal care workers, including nursing staff, is the main challenge facing the social welfare system in Poland.

**3. General evaluation of the content of article**

 The main objective of this article was to analyze the attitudes of care workers in nursing homes toward the reality of the pandemic in Poland, in four basic contexts: assessment of the degree of social care in the pandemic era; assessment of the most stressful factors since the outbreak of the pandemic; assessment of the mental wellbeing of care workers and evaluation of institutional measures taken to improve mental health. The issues raised are a very important topic, which is e.g. the results of the experience of the pandemic in recent years, as well as current problems related to the protection of people under residential care.

**4. Formal evaluation**

 The article is factually and methodologically correct and fits into the current discourse in the area of social work. The layout of the article is appropriate and structurally corresponds to articles presenting the results of empirical research. The applied terminology is part of research in the field of social sciences, with a particular focus on social work. The applied methodology of quantitative research, as well as the criteria for selecting a research sample correspond to the specificity of this type of research, creating space for their thematic and statistic interpretation and final reflection of results. The literature taking into account newer proposals in this field was used properly and allowed for the confrontation of the results with other studies in the discussion and summary.

**Final remarks**

 The conducted empirical research was to partially fill the gap in knowledge about the condition of care workers in Poland during the coronavirus pandemic. The proposed cultural approach, the experiences of care workers were considered as the important ones, because they were treated as a social mirror of the resourcefulness of institutions in the crisis caused by the pandemic. The issues raised from the research undoubtedly determine the importance of the problems raised in the context of resident care for dependent persons, but also the use of knowledge and experience from the pandemic period of employees of these institutions in securing adequate medical, psychological and social care and a support system corresponding to the needs of the wards. The research methodology was used correctly and allowed to formulate some proposals for social practice. The analysis of the data allowed to design and implement forms of support most suitable for the reported needs, based on the direct experiences of care workers. The organization of various forms of mental health support for formal care workers, including nursing staff, is the main challenge facing the social welfare system in Poland.

Is the work clearly and accurately presented and does it cite the current literature?

Yes

If applicable, is the statistical analysis and its interpretation appropriate?

Yes

Are all the source data underlying the results available to ensure full reproducibility?

Yes

Is the study design appropriate and is the work technically sound?

Yes

Are the conclusions drawn adequately supported by the results?

Yes

Are sufficient details of methods and analysis provided to allow replication by others?

Yes

Reviewer Expertise:

Social work theory, methodology of sw research, child protection, family support, profssionalization of social work, comparative social work.

I confirm that I have read this submission and believe that I have an appropriate level of expertise to confirm that it is of an acceptable scientific standard.
